# The abundance of the Lyme disease pathogen *Borrelia afzelii* declines over time in the tick vector *Ixodes ricinus*

**DOI:** 10.1186/s13071-017-2187-4

**Published:** 2017-05-25

**Authors:** Maxime Jacquet, Dolores Genné, Alessandro Belli, Elodie Maluenda, Anouk Sarr, Maarten J. Voordouw

**Affiliations:** Laboratory of Ecology and Evolution of Parasites, Institute of Biology, University of Neuchâtel, Neuchâtel, Switzerland

**Keywords:** *Borrelia afzelii*, Host-to-tick transmission, *Ixodes ricinus*, Lyme borreliosis, Pathogen burden, Spirochete load, Tick-borne disease, Vector-borne disease

## Abstract

**Background:**

The population dynamics of vector-borne pathogens inside the arthropod vector can have important consequences for vector-to-host transmission. Tick-borne spirochete bacteria of the *Borrelia burgdorferi* (*sensu lato*) species complex cause Lyme borreliosis in humans and spend long periods of time (>12 months) in their *Ixodes* tick vectors. To date, few studies have investigated the dynamics of *Borrelia* spirochete populations in unfed *Ixodes* nymphal ticks.

**Methods:**

Larval ticks from our laboratory colony of *I. ricinus* were experimentally infected with *B. afzelii*, and killed at 1 month and 4 months after the larva-to-nymph moult. The spirochete load was also compared between engorged larval ticks and unfed nymphs (from the same cohort) and between unfed nymphs and unfed adult ticks (from the same cohort). The spirochete load of *B. afzelii* in each tick was estimated using qPCR.

**Results:**

The mean spirochete load in the 1-month-old nymphs (~14,000 spirochetes) was seven times higher than the 4-month-old nymphs (~2000 spirochetes). Thus, the nymphal spirochete load declined by 80% over a period of 3 months. An engorged larval tick acquired ~100 spirochetes, and this population was 20 times larger in a young, unfed nymph. The spirochete load also appeared to decline in adult ticks. Comparison between wild and laboratory populations found that lab ticks were more susceptible to acquiring *B. afzelii*.

**Conclusion:**

The spirochete load of *B. afzelii* declines dramatically over time in domesticated *I. ricinus* nymphs under laboratory conditions. Future studies should investigate whether temporal declines in spirochete load occur in wild *Ixodes* ticks under natural conditions and whether these declines influence the tick-to-host transmission of *Borrelia*.

**Electronic supplementary material:**

The online version of this article (doi:10.1186/s13071-017-2187-4) contains supplementary material, which is available to authorized users.

## Background

Vector-borne pathogens reproduce, develop, and migrate inside their arthropod vector. The population dynamics of vector-borne pathogens inside their arthropod vectors plays a critical role in the transmission of vector-borne diseases. The number of pathogens inoculated by the arthropod vector into the vertebrate host is one of the key predictors of vector-to-host transmission success [1–3], and this variable often depends on the duration of vector feeding, which gives the pathogen population time to replicate and migrate to the relevant tissues [4, 5]. For many arboviruses, the speed at which mosquito vectors become infectious to their vertebrate hosts depends on the population growth rate of the virus inside the vector, which depends on extrinsic environmental factors such as temperature [2, 6, 7]. Studies of tick-borne pathogens that establish multiple-strain infections have found that the abundance of a particular strain in the arthropod vector is an important predictor of strain-specific transmission and fitness [8–10]. In summary, the study of the factors that influence the pathogen population size inside the arthropod vector may give us a better understanding of the epidemiology of vector-borne diseases [11].

*Borrelia burgdorferi* (*sensu lato*) (*s.l.*) is a species complex of spirochete bacteria that cause Lyme borreliosis (LB) in humans, and that are transmitted by *Ixodes* ticks [12–14]. Larval ticks acquire *Borrelia* spirochetes from infected vertebrate reservoir hosts and moult into infected nymphs, which transmit the pathogen to new hosts. The larval and nymphal blood meals occur in consecutive summers, and the *Borrelia* spirochetes spend 1 year or more in the immature tick [15–17]. How *Borrelia* spirochetes persist inside unfed *Ixodes* nymphs is currently unknown [18], but there is a lot of interest in identifying the underlying genes [18–24]. Studies that have characterised *Borrelia* population dynamics in *Ixodes* nymphs have mostly focused on the short duration of attachment to the vertebrate host (<7 days) and the resulting pathogen transmission [25–31]. In contrast, there are not many studies that have investigated spirochete population dynamics during the long sojourn inside unfed nymphal ticks. Early microscope studies on *B. burgdorferi* (*sensu stricto*) (*s.s*.) in *I. scapularis* found that the spirochete abundance in the nymphal tick following the larva-to-nymph moult was low (~300 spirochetes) [26, 32], suggesting that it was constant over time. In contrast, a recent study on the European LB pathogen, *B. afzelii*, in its tick vector, *I. ricinus*, observed that the spirochete population in the nymphal tick following the larva-to-nymph moult was high (>10,000 spirochetes) and that it declined dramatically (by almost 90%) over a period of 6 months [33]. This observed decline in spirochete load was a serendipitous discovery, and the study was not designed to answer that particular question [33]. The purpose of the present study is therefore to test whether the spirochete load of *B. afzelii* changes over time in unfed *I. ricinus* nymphs. The spirochete load was also investigated in engorged larval ticks and unfed adult ticks to obtain a better understanding of how the population size of *B. afzelii* inside *I. ricinus* changes over the life-cycle of the tick vector.

## Methods

### Strains of *Borrelia afzelii*

*Borrelia afzelii* isolates E61 and NE4049 were used in this study. These isolates have ID numbers 1888 and 1887 in the *Borrelia* MLST database, respectively. E61 was originally isolated from a human patient in Austria whereas NE4049 was isolated from an *I. ricinus* tick in Neuchâtel, Switzerland. Isolate E61 has multi-locus sequence type (MLST) ST75 and *ospC* major group (oMG) A3, whereas isolate NE4049 has ST679 and oMG A10. For simplicity and as we have done elsewhere, these two isolates will hereafter be referred to as *B. afzelii ospC* strains A3 and A10 [33–35]. We have previously characterised the co-feeding and systemic transmission phenotypes of these two *ospC* strains over the acute and chronic phase of the infection [33–36].

### Creation of *B. afzelii*-infected laboratory *I. ricinus* nymphs killed at 1 and 4 months post-moult

Female *Mus musculus* Balb/cByJ mice were experimentally infected with either *B. afzelii ospC* strain A3 or strain A10 *via* nymphal tick bite. The details of this infection experiment have been described elsewhere [33, 34, 36]. There were 10 and 13 mice that became systemically infected with strain A3 and strain A10, respectively. The mice were infested with larval *I. ricinus* ticks on five separate occasions at 2, 34, 66, 94, and 128 days post-infection (PI). For each infestation, 50–100 larvae were placed on each mouse. All ticks came from the pathogen-free, laboratory colony of *I. ricinus* at the University of Neuchâtel. Engorged larval ticks were placed in individual tubes and were allowed to moult into nymphs [33, 34, 36]. Larval and nymphal ticks were kept under controlled laboratory conditions: the temperature was 22 °C and the relative humidity was > 80%. The present study only concerns the larval ticks from the last three infestations (66, 94, and 128 days PI). For each of the 69 combinations of mouse (*n* = 23 mice) and infestation (66, 94, and 128 days PI), we randomly selected a maximum of 10 nymphs and froze them at -20 °C at 1 and 4 months after the larva-to-nymph moult.

### Spirochete load of *B. afzelii* in engorged larvae and flat nymphs

To determine how the abundance of *B. afzelii* changes over the course of the life-cycle of *I. ricinus*, the spirochete loads were compared between engorged larval ticks and flat nymphs. Each of 10 female Balb/cByJ mice was experimentally infected with *B. afzelii ospC* strain A10 *via* nymphal tick bite. Four weeks after the nymphal challenge, *B. afzelii* infection in the mice was confirmed with a *Borrelia*-specific qPCR assay on an ear tissue sample and a commercial ELISA to detect *Borrelia*-specific IgG antibodies. Five weeks after the nymphal challenge, each mouse was challenged with ~100 pathogen-free xenodiagnostic larvae from our laboratory colony of *I. ricinus*. Blood-engorged larvae were placed in individual Eppendorf tubes, and 5 engorged larvae were randomly selected for each mouse and frozen at -20 °C within 48 h of drop-off. The remaining engorged larval ticks were allowed to moult into nymphs under controlled laboratory conditions. At 4 weeks after the larva-to-nymph moult, 10 live nymphs were randomly selected for each mouse and frozen at -20 °C.

### Spirochete load of *B. afzelii* in flat nymphs and flat adult ticks

To determine how the abundance of *B. afzelii* changes over the course of the life-cycle of *I. ricinus*, the spirochete loads were compared between flat nymphs and flat adult ticks. Each of 7 mice infected *via* nymphal tick bite with *B. afzelii ospC* strain A10 was infested with larval ticks from our laboratory colony of *I. ricinus* at 30 days post-infection. The engorged larval ticks were allowed to moult into nymphs. To test for the prevalence of *B. afzelii* infection, 5 nymphs were randomly sampled from each mouse and frozen at -20 °C at 4 weeks after the larva-to-nymph moult. At ~170 days after the larval blood meal and ~90 days after the larva-to-nymph moult, 100 of these nymphal ticks were fed on 20 pathogen-free, female Balb/cByJ mice (5 nymphs/mouse). Nymphs were placed in plastic capsules that were attached to the backs of the mice, as we have described previously [33]. The engorged nymphal ticks were kept in individual Eppendorf tubes under standard conditions and were allowed to moult into adult ticks. The adult ticks were killed on a date that corresponded to the following times in the tick life-cycle: 198 days (28 weeks) after the nymph-to-adult moult, 271 days (39 weeks) after the nymphal blood meal, and 362 days (52 weeks) after the larva-to-nymph moult.

### Creation of *B. afzelii*-infected wild *I. ricinus* nymphs frozen at 1 month post-moult

Wild *I. ricinus* larval ticks were collected from a field site near Neuchâtel, Switzerland in the summer of 2014. Ten female Balb/cByJ mice were experimentally infected with isolate NE4049 via nymphal tick bite. At 5 weeks PI, infection of the mice was confirmed using a commercial Lyme disease ELISA. At 6 weeks PI, batches of 100 wild larvae were allowed to feed on the experimentally infected mice. Engorged larval ticks were collected and stored under controlled laboratory conditions as above. For each of the 10 mice, 10 nymphs were randomly selected and frozen at -20 °C at 1 month after the larva-to-nymph moult.

### DNA extraction of nymphal ticks and qPCR to determine *B. afzelii* infection

Total DNA was extracted from individual ticks using a TissueLyser II and DNeasy 96 Blood & Tissue kit well plates (Qiagen, Hilden, Germany). The DNA extraction protocol was described in a previous study [33]. A quantitative PCR amplifying a 132 bp fragment of the *flagellin* gene [37] was used to detect and quantify *Borrelia* DNA. The qPCR protocol was described in a previous study [33]. For the 1-month-old ticks in the first experiment, we conducted three replicate qPCR runs for each tick. The repeatability of the log10-transformed spirochete load estimated by these three replicate runs was very high (*r* = 0.972) [33]. We therefore ran single qPCR runs for the other ticks.

### Statistical analysis

All statistical analyses were done in R version 3.1.0. [38]. The datasets generated and analysed in the present study are available in Additional file 1.

#### Paired data for 1-month-old nymphs and 4-month-old nymphs

In the first experiment, paired data were obtained on 1-month-old nymphs and 4-month-old nymphs for 43 of the 69 possible combinations of mouse (*n* = 23 mice) and infestation (66, 94, and 128 days PI). The data are paired because the 1-month-old nymphs and the 4-month-old nymphs fed as larvae on the same mouse during the same infestation. The only difference between these two groups of nymphs is that they were killed at different times following the larva-to-nymph moult.

#### Definition of *B. afzelii* infection status for nymphal ticks

The nymphs were considered as infected with *B. afzelii* if the nymphal spirochete load estimated by the qPCR was > 1. For the first experiment, the proportions of infected ticks for the 1-month-old nymphs and the 4-month-old nymphs were calculated for each of the 43 available combinations of mouse and infestation.

#### Spirochete loads of *B. afzelii*-infected nymphal ticks

The total spirochete load for each nymph was calculated by adjusting the spirochete estimate for 5 μl of DNA template of the qPCR reaction to the volume of the DNA extraction (65 μl). As gene copy data from qPCR assays follow an exponential distribution, the spirochete loads were log10-transformed to improve their fit to the normal distribution. In the first experiment, the spirochete load of each 1-month-old nymph was the average of the three replicate qPCR runs (negative runs were excluded). The spirochete load of each 4-month-old nymph and each tick in the other experiments was the estimate from the single qPCR run. For each of the 43 infestations in the first experiment, the mean nymphal spirochete load was calculated separately for the 1-month-old nymphs and the 4-month-old nymphs using the subset of infected ticks (negative ticks were excluded).

#### Effect of time on the nymphal spirochete load of *B. afzelii*

Means and standard errors of the spirochete load were calculated on the log10-transformed scale before being back-transformed to the original scale. For the first experiment, a paired *t*-test was used to compare the mean proportion of infected ticks and the mean log10-transformed spirochete load between the 1-month-old nymphs and the 4-month-old nymphs. For the first experiment, Pearson’s correlation was used to test whether the proportion of infected ticks and the log10-transformed spirochete load were correlated between the 1-month-old nymphs and the 4-month-old nymphs.

## Results

### Effect of nymphal age on the probability of detecting *B. afzelii* in nymphal ticks

The mean proportion of infected nymphs was similar between the 1-month-old nymphs (61.8% = 259/419) and the 4-month-old nymphs (64.3% = 173/269) and the difference was not statistically significant (*t* = 0.695, *df* = 42, *P* = 0.491).

### Effect of nymphal age on the spirochete load of infected nymphs

The mean spirochete load of the 1-month-old nymphs (*n* = 43, mean: 14,094, 95% confidence interval (CI): 13,508–14,705 spirochetes per nymph) was 7.05 times larger than that of the 4-month-old nymphs (*n* = 42, mean: 1999, 95% CI: 1900–2103 spirochetes per nymph) and this difference was highly significant (*t* = 12.768, *df* = 41, *P* < 0.0001). The mean spirochete load of the 1-month-old nymphs was higher than that of the 4-month-old nymphs for 40 of the 42 pairs for which there were data.

**Fig. 1:**
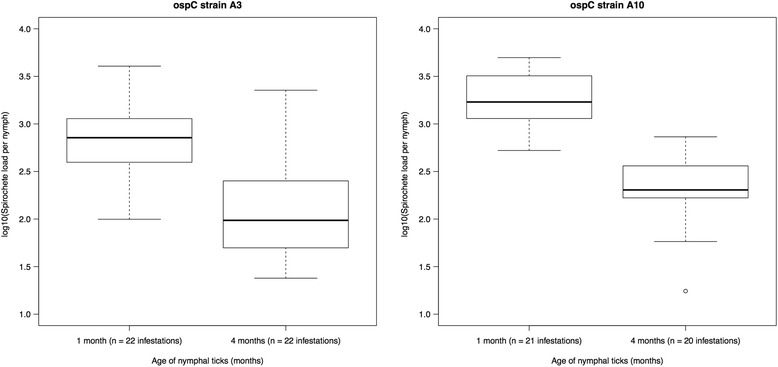
The spirochete loads of *B. afzelii* in *I. ricinus* nymphal ticks decreased over time. The panels show two different strains of *B. afzelii*: A3 and A10. For each strain, the spirochete load was compared between nymphs that are 1 and 4 months old. A3- or A10-infected mice were infested with larval ticks on 22 and 21 occasions, respectively (total of 43 infestations). The engorged larval ticks from these infestations were allowed to moult into nymphs and were killed at 1 or 4 months after the larva-to-nymph moult. The spirochete loads of the 1-month-old nymphs and the 4-month-old nymphs therefore represent paired data. Each data point is a mean of the subset of infected nymphs (i.e. uninfected nymphs were excluded). The spirochete loads in the nymphs were estimated using a qPCR that targeted a 132 bp fragment of the *flagellin* gene. The spirochete loads were log10-transformed to improve their fit to the normal distribution. Shown are the medians (*black line*), the 25th and 75th percentiles (*edges of the box*), the minimum and maximum values (*whiskers*), and the outliers (*solid circles*)

For strain A3, the mean spirochete load of the 1-month-old nymphs (*n* = 22, mean: 8880, 95% CI: 8169–9654 spirochetes per nymph) was 5.8 times larger than the 4-month-old nymphs (*n* = 22, mean: 1525, 95% CI: 1367–1700 spirochetes per nymph) and this difference was significant (*t* = 7.804, *df* = 21, *P* < 0.0001; Fig. 1). For strain A3, the flat nymphs lost an average of 81.7 spirochetes per day. For strain A10, the mean spirochete load of the 1-month-old nymphs (*n* = 21, mean: 22,865, 95% CI: 21,444–24,380 spirochetes per nymph) was 8.5 times larger than that of the 4-month-old nymphs (*n* = 20, mean: 2693, 95% CI: 2459–2949 spirochetes per nymph) and this difference was significant (*t* = 10.825, *df* = 19, *P* < 0.0001; Fig. 1). For strain A10, the flat nymphs lost an average of 208.3 spirochetes per day.

### Correlations in phenotype between young and old nymphs

**Fig. 2:**
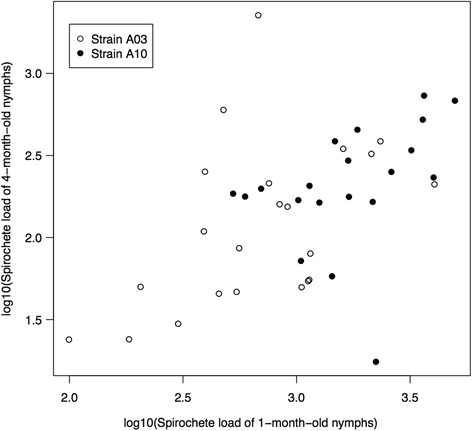
The log10-transformed nymphal spirochete loads of *B. afzelii* are highly correlated between the 1-month-old and the 4-month-old *I. ricinus* nymphs. Strains A3 and A10 are shown with *white* and *black* circles, respectively. A3- or A10-infected mice were infested with larval ticks on 43 different occasions. The engorged larval ticks from these infestations were allowed to moult into nymphs and were killed at 1 or 4 months after the larva-to-nymph moult. The spirochete loads of the 1-month-old nymphs and the 4-month-old nymphs therefore represent paired data. Each data point is a mean of the subset of infected nymphs (i.e. uninfected nymphs were excluded) that originated from the same infestation event. The spirochete loads in the nymphs were estimated using a qPCR that targeted a 132 bp fragment of the *flagellin* gene

The proportion of infected nymphs was not correlated between the 1-month-old nymphs and the 4-month-old nymphs (*r* = 0.161, *t* = 1.046, *df* = 41, *P* = 0.302). In contrast, the mean log10-transformed spirochete load was highly correlated between the 1-month-old nymphs and the 4-month-old nymphs (*r* = 0.510, *t* = 3.745, *df* = 40, *P* < 0.001). After removing the 10 pairs for which less than five 4-month-old nymphs were recovered, the magnitude of the correlation of the log10-transformed spirochete load increased (*r* = 0.580, *t* = 3.966, *df* = 31, *P* < 0.001; Fig. 2).

### Spirochete load of *B. afzelii* in engorged larvae and flat nymphs

**Fig. 3:**
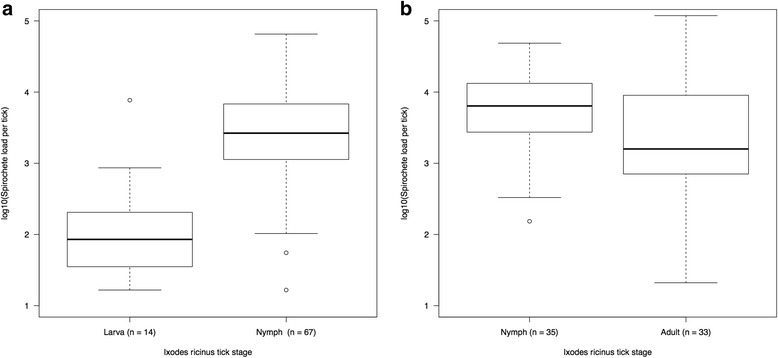
The spirochete load of *B. afzelii* in the tick changes over the life-cycle of *I. ricinus*. **a** The log10-transformed spirochete load increased 20-fold from the engorged larva (48 h after drop-off) to the nymph (4 weeks after the larva-to-nymph moult). **b** The log10-transformed spirochete load decreased 3-fold from the nymph (4 weeks after the larva-to-nymph moult) to the adult (28 weeks after the nymph-to-adult moult). The spirochete loads in the ticks were estimated using a qPCR that targeted a 132 bp fragment of the *flagellin* gene. The spirochete loads were log10-transformed to improve their fit to the normal distribution. Shown are the medians (*black line*), the 25th and 75th percentiles (*edges of the box*), the minimum and maximum values (*whiskers*), and the outliers (*solid circles*)

Of the 46 engorged larvae that were randomly sampled, 30.4% (14/46) tested positive for *B. afzelii*. The remaining engorged larvae from the same infestation were allowed to moult into nymphs. Of the 86 nymphs that were randomly sampled, 77.9% (67/86) tested positive for *B. afzelii*. The proportion of infected ticks increased 2.6-fold from the engorged larvae (tested at 48 h after drop-off) to the flat nymphs (tested at 4 weeks after the larva-to-nymph moult), and this difference was significant (*χ* ^2^ = 26.52, *df* = 1, *P* < 0.001). The mean spirochete load for the subset of *B. afzelii*-infected engorged larvae (*n* = 14, mean: 123, 95% CI: 53–285 spirochetes per engorged larva) was 20 times smaller than that of the subset of *B. afzelii*-infected nymphs (*n* = 67, mean: 2416, 95% CI: 1646–3546 spirochetes per nymph) and this difference was significant (*t* = 6.420, *df* = 79, *P* < 0.001; Fig. 3a).

### Spirochete load of *B. afzelii* in flat nymphs and flat adult ticks

Of the pool of nymphs used to create the adult ticks, 35 nymphs were randomly selected, and 100.0% (35/35) tested positive for *B. afzelii*. Of the remaining nymphs, 100 were fed on 20 uninfected mice, and 82 engorged nymphs were recovered, of which 62 moulted into adult ticks. Of these 62 adult ticks, 53.2% (33/62) tested positive for *B. afzelii*. The proportion of infected ticks decreased 1.9-fold from the flat nymphs (tested at 4 weeks after the larva-to-nymph moult) to the flat adults (tested at 28 weeks after the nymph-to-adult moult), and this difference was significant (*χ* ^*2*^ = 21.17, *df* = 1, *P* < 0.001). The percentage of *B. afzelii*-positive ticks was the same between males (58.1% = 18/31) and females (48.4% = 15/31). The mean spirochete load for the subset of *B. afzelii*-infected nymphs (*n* = 35, mean: 5801, 95% CI: 3288–10,234 spirochetes per nymph) was 3 times larger than that for the subset of *B. afzelii*-infected adults (*n* = 33, mean: 1959, 95% CI: 1092–3516 spirochetes per adult tick) and this difference was significant (*t* = 2.659, *df* = 79, *P* < 0.010; Fig. 3b). There was no effect of sex on the mean spirochete load in the adult ticks.

### Mean spirochete load in wild *I. ricinus* nymphs

Of the 100 wild *I. ricinus* nymphs that were frozen at 1 month post-molt, 69 were infected with *B. afzelii ospC* strain A10. The mean spirochete load for the subset of *B. afzelii*-infected nymphs was 2793 spirochetes per nymph (*n* = 69, 95% CI: 1761–4492 spirochetes per nymph).

## Discussion

The population of *B. afzelii* spirochetes in *I. ricinus* nymphal ticks declined dramatically over time (Fig. 1). Over a period of 3 months, the mean nymphal spirochete load declined by 82.8 and 88.2% for strains A3 and A10, respectively. This result was similar to a pilot study on the same system that compared the mean spirochete load between 1-month-old nymphs and 7-month-old nymphs [33]. In that study, the nymphal spirochete load declined by 47.2 and 86.5% for strains A3 and A10, respectively [33]. The sample size in the present study (432 infected nymphs) is much larger than the pilot study (80 infected nymphs). The paired experimental design used in the present study also provided a more powerful statistical test because it controlled for substantial variation in mean spirochete load between larval infestations (Fig. 2). We are not aware of any other studies on the dynamics of the spirochete population size over time inside unfed nymphal ticks after the larva-to-nymph moult. Early microscope studies on *B. burgdorferi* (*s.s*.) in *I. scapularis* quantified the nymphal spirochete load shortly after the larva-to-nymph moult [32] or at a single unspecified time point [26]. The estimates of the nymphal spirochete loads in these microscope-based studies are much lower than the present study. For example, Piesman et al. [32] estimated that *I. scapularis* nymphs contain < 300 *B. burgdorferi* (*s.s*.) spirochetes per tick at 3 to 12 days after the larva-to-nymph moult. Similarly, De Silva & Fikrig [26] estimated that nymphs contain 496 spirochetes per tick at some unspecified time point after the larva-to-nymph moult. In contrast, the present study found that *I. ricinus* nymphs contained a mean of 8880 or 14,094 *B. afzelii* spirochetes per tick at four weeks after the larva-to-nymph moult depending on the strain. The large differences in nymphal spirochete load between the early studies and the present study might be due to differences in the *Borrelia* species, the tick species, or the method of quantification (microscopy *versus* qPCR).

Why does the spirochete population size inside the unfed nymphal tick decline over time? One possible explanation is that digestion of the larval blood meal in the midgut of the unfed nymph reduces the spirochete population over time [18]. Previous work on *B. burgdorferi* (*s.s*.) in *I. scapularis* and on *B. burgdorferi* (*s.l*.) in *I. ricinus* has shown that the spirochete population in the unfed nymph is predominantly located in the tick midgut [26, 39–42]. Molecular studies have identified the outer surface proteins (e.g. OspA and OspB) that *Borrelia* pathogens use to anchor themselves to the tick midgut [21–24]. Digestion of the blood meal in ticks is a slow process that takes place within the epithelial cells lining the tick midgut [43, 44]. Endocytosis and intracellular digestion of the blood meal by epithelial cells could also result in the ingestion and destruction of spirochetes [18]. There is indirect evidence suggesting that digestion of the larval blood meal continues in unfed nymphs. First, proteomics analysis has detected residual blood proteins from the vertebrate host in unfed nymphs at 3–11 months post-moult [45, 46]. Second, host blood meal studies have shown that the ability to detect and identify host DNA in unfed nymphs decreases over time [47, 48]. These observations suggest that the digestion of host proteins and host DNA from the larval blood meal continues in unfed nymphs. Thus one explanation for the temporal decline in the spirochete load is the slow but continuous digestion of the larval blood meal and the spirochete population in the unfed nymphal tick.

Does the temporal decline in spirochete load in the nymphal tick influence tick-to-host transmission of the *Borrelia* pathogen? During the nymphal blood meal, the spirochetes divide in the tick midgut and migrate to the tick salivary glands [26, 41, 42]. This spirochete migration inside the tick explains why the probability of infection increases with the duration of attachment to the host [5, 25, 26, 30, 31]. A recent study on *B. burgdorferi* (*s.s*.) in *I. scapularis* nymphs found that nymphal age and temperature treatment had no effect on the spirochete load in the engorged nymph [20]. For example, nymphs that were aged for 0 months, 3 months (at 4 °C) or 9 months (3 months at 4 °C followed by 6 months at room temperature) all had the same spirochete load following engorgement [20]. This study suggests that spirochete replication following tick attachment might compensate for any temporal decline in nymphal spirochete load. However, other studies suggest that the nymphal spirochete load may influence strain-specific transmission to the vertebrate host. A study on *B. afzelii ospC* strains in a wild *I. ricinus* population found that the strains that established the highest spirochete load in the flat nymphs were the most common in the tick population [10]. A study using genetically tagged strains of *B. burgdorferi* (*s.s*.) found that the strains with the highest spirochete load in the nymphal tick had a higher probability of being transmitted to the rodent host [8]. These two studies suggest that the spirochete load in the nymphal tick is an important life history trait of *Borrelia* pathogens. Future studies should investigate whether the temporal decline in the nymphal spirochete load influences the tick-to-host transmission success of *Borrelia* pathogens.

Does the temporal decline in spirochete load influence our ability to detect *Borrelia* infection in nymphal ticks? In the first experiment, the proportion of infected ticks did not change between the 1-month-old nymphs and the 4-month-old nymphs. This result demonstrates that the sensitivity of our qPCR assay is high and that the probability to detect *B. afzelii* infection in *I. ricinus* nymphs was not affected by a seven-fold drop in spirochete load. In contrast, a study on *B. burgdorferi* (*s.s*.) in *I. scapularis* nymphs using conventional PCR found that the proportion of infected ticks declined from 74.0 to 15.5% between 1-month-old nymphs and 5-month-old nymphs [49]. One interpretation of these data is that the conventional PCR was unable to detect the infection once the spirochete load dropped below a certain threshold. A limitation with PCR-based methods is that we cannot distinguish between spirochetes that are dead or alive. Future investigations should use methods that allow us to assess microbial viability [50].

The present study showed that the population of *B. afzelii* spirochetes increased dramatically from the engorged larval tick to the flat nymphal tick (Fig. 3a). A larval tick acquired about 100 spirochetes after feeding on an infected mouse, and this spirochete population grew exponentially so that it was 20 times larger in the nymph at 4 weeks after the larva-to-nymph moult. The early microscope study on *B. burgdorferi* (*s.s*.) in *I. scapularis* by Piesman et al. [32] showed a similarly dramatic expansion of the spirochete population inside the engorged larval tick over the first 15 days post-repletion. The study by Piesman et al. [32] did not estimate the number of spirochetes acquired by a feeding larval tick, but extrapolation of their data suggests that the inoculum size is < 250 spirochetes. The observation in the present study that the proportion of infected ticks increased 2.6-fold from the engorged larval ticks to the flat nymphs is an issue of detection and false negatives. In some engorged larval ticks, the number of spirochetes acquired was so low that our qPCR assay did not detect them. Another explanation for the low ability to detect *B. afzelii* infections in engorged larvae is that heme in blood is a known inhibitor of PCR [51, 52]. The larval blood meal is an important bottleneck that determines the genetic diversity of the *Borrelia* population that is acquired by the larval tick and subsequently transmitted by the resultant nymph [8]. Thus accurate estimates of how many spirochetes are acquired by a larval tick are important for understanding the population genetics of *Borrelia* spirochetes [53].

Our study found that the spirochete load in the unfed adult ticks was three times lower than that in the unfed nymphal ticks (Fig. 3b). Numerous studies have shown that the spirochete load inside the nymph increases dramatically during the nymphal blood meal [20, 26–28, 32, 33, 54, 55]. For example, the mean spirochete load of 7-month-old flat nymphs infected with *B. afzelii ospC* strains A3 and A10 (743 and 1537 spirochetes), increased by a factor of 4.8 and 1.9, respectively, when the nymphs engorged on the laboratory mice (3530 and 2896 spirochetes) 4 months later [33]. In those studies, the engorged nymphs were killed during or shortly after completing the blood meal. In the present study, by contrast, the adult ticks were killed at 198 days after the nymph-to-adult moult. If adult ticks lose spirochetes at the same rate as observed for nymphs in the present study (82–208 spirochetes per day), we would expect some of the spirochete populations to go extinct after 198 days of blood meal digestion. Thus, one explanation for the observation that the prevalence of *B. afzelii* declined from 100.0% in the flat nymphs to 53.2% in the flat adults was that the spirochete population had decreased below the qPCR detection threshold. Life history theory suggests that selection is weak for *Borrelia* pathogens to maintain a viable spirochete population in adult ticks. Adult ticks are dead-end vectors for *Borrelia* because the males do not blood feed and because the females tend to feed on incompetent reservoir hosts such as deer [56–58]. We therefore expect *Borrelia* spirochete populations to decline in quantity and quality over time inside adult ticks. Future studies should investigate whether the observed decline in spirochete load in the nymphal ticks also occurs in the adult ticks.

Is the spirochete load of *B. afzelii* in our laboratory population of *I. ricinus* ticks similar to what is observed in wild ticks? Our laboratory population of *I. ricinus* was domesticated at the University of Neuchâtel almost 40 years ago [59]. It is possible that laboratory selection has changed the susceptibility of our laboratory population of *I. ricinus* to infection with *B. afzelii*. We therefore collected wild *I. ricinus* larva at a field site near Neuchâtel, fed them on lab mice infected with *B. afzelii* isolate NE4049 (*ospC* strain A10), and tested the nymphs at 1 month post-moult. The prevalence of *B. afzelii* infection was 1.4 times higher for the lab nymphs than the wild nymphs. This observation suggests that lab ticks are more susceptible to acquire *B. afzelii* infection than wild nymphs. The mean spirochete load in these 1-month-old wild nymphs was 8.2 times lower than the 1-month-old lab nymphs but similar to that of the 4-month-old lab nymphs. This observation suggests that the laboratory ticks are less capable of controlling the *B. afzelii* spirochete load than the wild nymphs.

In addition, we recently quantified the *Borrelia* spirochete load in field-collected unfed *I. ricinus* nymphs [60]. Most of these nymphs were collected in the spring and would have fed as larvae the previous summer or fall, suggesting that the *Borrelia* infections were 6 to 9 months old. For the 788 wild *I. ricinus* nymphs that were infected with *B. afzelii*, the geometric mean spirochete load (2003 spirochetes per nymph) [60] was similar to that observed for the 4-month-old nymphs (1999 spirochetes per nymph; averaged across the two strains) in the present study. Taken together, these studies suggest that the high *B. afzelii* spirochete loads observed in the 1-month-old lab nymphs may be a phenotype that is specific to our laboratory population of *I. ricinus*. Future studies should investigate whether the observed declines in *Borrelia* spirochete load occur in wild ticks under natural conditions.

## Conclusions

In the present study, we showed that the abundance of *B. afzelii* in unfed *I. ricinus* nymphs declines by as much as 90% over a period of 3 months when nymphs were kept under laboratory conditions. Researchers interested in quantifying the spirochete load in ticks should be aware that this phenotype declines dramatically over time. Future studies should investigate whether the *Borrelia* spirochete load declines over time in wild *Ixodes* ticks under natural conditions and whether these changes in spirochete load influence the tick-to-host transmission success of *Borrelia* pathogens.

## Additional file

Additional file 1:
**Table S1.** List of definitions for the variables that appear in the column headings of Tables S2, S3 and S4. **Table S2.** Raw data of the *Borrelia afzelii *spirochete loads for the 1-month-old *Ixodes ricinus* nymphs and the 4-month-old *I. ricinus* nymphs used to create Figs. 1 and 2. **Table S3.** Raw data of the *B. afzelii *spirochete loads for the engorged larval ticks and the resultant flat nymphs used to create Fig. 3a and the raw data of the *B. afzelii* spirochete loads for the flat nymphs and the resultant adult ticks used to create Fig. 3b. **Table S4. **Raw data of the *B. afzelii* spirochete loads for the wild *I. ricinus* nymphs. (XLSX 96 kb)

## Abbreviations

ELISA: Enzyme-linked immunosorbent assay
LB: Lyme borreliosis
OspC: Outer surface protein C
qPCR: Quantitative real-time polymerase chain reaction.
